# Annexin A1 protects against cerebral ischemia–reperfusion injury by modulating microglia/macrophage polarization via FPR2/ALX-dependent AMPK-mTOR pathway

**DOI:** 10.1186/s12974-021-02174-3

**Published:** 2021-05-22

**Authors:** Xin Xu, Weiwei Gao, Lei Li, Jiheng Hao, Bin Yang, Tao Wang, Long Li, Xuesong Bai, Fanjian Li, Honglei Ren, Meng Zhang, Liyong Zhang, Jiyue Wang, Dong Wang, Jianning Zhang, Liqun Jiao

**Affiliations:** 1 Department of Neurosurgery, Xuanwu Hospital, Capital Medical University, 45 Changchun Street, Beijing, 100053 China; 2 China International Neuroscience Institute (China-INI), 45 Changchun Street, Beijing, 100053 China; 3 Department of Neurology, Tianjin Huanhu Hospital, 6 Jizhao Road, Tianjin, 300350 China; 4 Department of Neurosurgery & Neurology, Tianjin Medical University General Hospital, 154 Anshan Road, Tianjin, 300052 China; 5 Department of Neurosurgery, Liaocheng People’s Hospital, 67 Dongchang West Road, Liaocheng, 252000 China; 6 Department of Interventional Neuroradiology, Xuanwu Hospital, Capital Medical University, 45 Changchun Street, Beijing, 100053 China

**Keywords:** Cerebral ischemia-reperfusion injury, Endovascular thrombectomy, Neuroinflammation, Microglial/macrophage polarization, Annexin A1, Formyl peptide receptor 2

## Abstract

**Background:**

Cerebral ischemia–reperfusion (I/R) injury is a major cause of early complications and unfavorable outcomes after endovascular thrombectomy (EVT) therapy in patients with acute ischemic stroke (AIS). Recent studies indicate that modulating microglia/macrophage polarization and subsequent inflammatory response may be a potential adjunct therapy to recanalization. Annexin A1 (ANXA1) exerts potent anti-inflammatory and pro-resolving properties in models of cerebral I/R injury. However, whether ANXA1 modulates post-I/R-induced microglia/macrophage polarization has not yet been fully elucidated.

**Methods:**

We retrospectively collected blood samples from AIS patients who underwent successful recanalization by EVT and analyzed ANXA1 levels longitudinally before and after EVT and correlation between ANXA1 levels and 3-month clinical outcomes. We also established a C57BL/6J mouse model of transient middle cerebral artery occlusion/reperfusion (tMCAO/R) and an *in vitro* model of oxygen–glucose deprivation and reoxygenation (OGD/R) in BV2 microglia and HT22 neurons to explore the role of Ac2-26, a pharmacophore N-terminal peptide of ANXA1, in regulating the I/R-induced microglia/macrophage activation and polarization.

**Results:**

The baseline levels of ANXA1 pre-EVT were significantly lower in 23 AIS patients, as compared with those of healthy controls. They were significantly increased to the levels found in controls 2–3 days post-EVT. The increased post-EVT levels of ANXA1 were positively correlated with 3-month clinical outcomes. In the mouse model, we then found that Ac2-26 administered at the start of reperfusion shifted microglia/macrophage polarization toward anti-inflammatory M2-phenotype in ischemic penumbra, thus alleviating blood–brain barrier leakage and neuronal apoptosis and improving outcomes at 3 days post-tMCAO/R. The protection was abrogated when mice received Ac2-26 together with WRW4, which is a specific antagonist of formyl peptide receptor type 2/lipoxin A4 receptor (FPR2/ALX). Furthermore, the interaction between Ac2-26 and FPR2/ALX receptor activated the 5’ adenosine monophosphate-activated protein kinase (AMPK) and inhibited the downstream mammalian target of rapamycin (mTOR). These *in vivo* findings were validated through *in vitro* experiments.

**Conclusions:**

Ac2-26 modulates microglial/macrophage polarization and alleviates subsequent cerebral inflammation by regulating the FPR2/ALX-dependent AMPK-mTOR pathway. It may be investigated as an adjunct strategy for clinical prevention and treatment of cerebral I/R injury after recanalization. Plasma ANXA1 may be a potential biomarker for outcomes of AIS patients receiving EVT.

**Supplementary Information:**

The online version contains supplementary material available at 10.1186/s12974-021-02174-3.

## Background

Ischemic stroke, which accounts for ~ 80% of all strokes, is a devastating cerebrovascular disease that brings high mortality and disability worldwide [1]. For patients with acute ischemic stroke (AIS), the primary therapeutic aim is timely and effective recanalization of the occluded blood vessels by thrombolytic therapy or endovascular thrombectomy (EVT) [1, 2]. EVT, alone or in combination with intravenous thrombolysis (bridging strategy), achieves > 80% of recanalization and markedly improves clinical outcomes of AIS patients caused by large vessel occlusion (LVO) to proximal anterior circulation [3]. However, many AIS patients who achieved successful recanalization develop early complications (e.g., secondary infarct growth, symptomatic hemorrhagic transformation, and malignant edema), and ~ 50% of these patients remain disabled several months post-EVT. This condition is now termed as “futile recanalization” [4–6]. It has been widely recognized that rapid reperfusion itself induced cerebral ischemia–reperfusion (I/R) injury is a major cause of futile recanalization [7–9]. However, therapeutic options for cerebral I/R injury prevention remain limited and are often ineffective, indicating the lack of understanding the pathophysiological mechanisms of cerebral I/R injury and targeted therapeutics [9, 10].

Accumulating evidence suggests that innate immunity and inflammatory response, especially the brain resident microglia and infiltrating macrophages, contribute to the cerebral I/R injury [10–12]. These cells are activated and recruited to the lesion site, primarily in the peri-infarct region, where these microglia/macrophages can be phenotypically polarized into two major phenotypes: the “classically activated” M1 and “alternatively activated” M2. M1 cells express signature markers, cluster of differentiation (CD)16, CD32, and CD86, and release pro-inflammatory mediators such as inducible nitric oxide synthase (iNOS) and interleukin (IL)-1β that aggravate brain damage. In contrast, M2 cells are characterized by the expression of signature markers of CD206, arginase-1 (Arg-1), and Ym1, and secretion of anti-inflammatory mediators such as IL-4 and IL-10 that promote tissue repair [11, 12]. The dysregulation of M1/M2 cells and their ratio contribute to the post-I/R cerebral inflammatory responses. It has been well studied that pro-inflammatory M1-phenotype increases rapidly on day 3 and dominates the peri-infarct region in the first week of cerebral I/R injury [12, 13]. Hence, exploring novel immunomodulation that balance microglia/macrophage phenotypes within the acute ischemic penumbra may provide a viable strategy to prevent or reduce cerebral I/R injury.

Annexin A1 (ANXA1, formerly lipocortin-1) is a 37-kDa glucocorticoid-regulated calcium- and phospholipid-binding protein. It has potent anti-inflammatory and pro-resolving activities that are mediated through the G-protein-coupled formyl peptide receptor type 2 (also known as the lipoxin A4 receptor; FPR2/ALX) [14]. Preclinical studies reveal that ANXA1 (endogenous or exogenous ANXA1, or its biologically active N-terminal domain termed Ac2-26)-FPR2/ALX interaction limits inflammation and attenuates tissue damage in the setting of splanchnic (e.g., kidney and lung) [15, 16], myocardial [17], and cerebral I/R injuries [18–20]. Luo et al. [21] reported that Ac2-26 drives microglia into an anti-inflammatory M2-phenotype and thus protecting neurons subjected to the oxygen–glucose deprivation and reoxygenation (OGD/R) *in vitro*. However, whether ANXA1 and Ac2-26 mediated phenotypic shift of microglia can be translated to an *in vivo* cerebral I/R situation, and its underlying mechanisms have not yet been fully elucidated. Ac2-26, the pharmacophore of ANXA1, has been proven to mimic various biological functions of ANXA1, making it a suitable candidate for mechanism research of ANXA1 [14]. More importantly, Ac2-26 may have a better prospect of clinical application than genetic approaches [22]. In this study, we used a well-established mouse model of transient middle cerebral artery occlusion/reperfusion (tMCAO/R) and an *in vitro* OGD/R model to study the role of Ac2-26 in I/R-induced microglia/macrophage activation and polarization, and the subsequent inflammatory injuries. In addition, few biomarkers have been reported to predict the unfavorable outcome/futile recanalization after EVT [6]. We therefore measured dynamic changes of plasma levels of ANXA1 in longitudinal samples from patients with AIS who received successful recanalization in order to determine whether ANXA1 predicts the unfavorable outcome/futile recanalization of AIS patients after EVT.

## Methods

### Patients

Consecutive AIS patients with first-ever acute LVO and achieved successful recanalization by EVT were retrospectively screened for eligibility of this study from May 22, 2019 to August 13, 2020 at the Liaocheng People’s Hospital (Liaocheng, China). Inclusion criteria were patients who underwent EVT with (1) age ≥ 18 years; (2) LVO of the anterior circulation including internal carotid artery (ICA), proximal MCA, or tandem occlusion; (3) groin puncture initiated within 6 h from symptom onset (or last known normal); and (4) successful recanalization defined as modified Treatment in Cerebral Infarction (mTICI) of 2b-3. Exclusion criteria were EVT-treated patients with (1) posterior circulation stroke; (2) pre-stroke modified Rankin Scale (mRS) ≥ 2 (pre-stroke disability); (3) unfavorable outcomes occurred before sampling; (4) concurrent or recent infection; (5) ongoing anti-inflammatory/immunosuppressant drug treatment; (6) malignant tumor; (7) severe liver or kidney failure; (8) hematological, rheumatic, or immune disorder; and (9) missing clinical/follow-up data. Demographic and clinical information were extracted from electronic medical records. In addition, twelve age- and sex-matched healthy individuals from the Xuanwu Hospital, Capital Medical University (Beijing, China) were also recruited as controls. Plasma levels of ANXA1 collected on admission and on the 2nd–3rd day post-EVT were measured by enzyme-linked immunosorbent assay (ELISA). Functional outcomes at 3 months after the onset of symptoms were assessed by mRS, and favorable clinical outcome was defined as mRS of 0 to 2. This human subject study was approved by the Ethics Committees of Liaocheng People’s Hospital and Xuanwu Hospital, Capital Medical University. All participants (or legal representatives) were informed of the study protocol and signed the consent form in accordance with the Helsinki declaration.

### Animals, tMCAO/R model, experimental design, and drug administration

Male C57BL/6J mice (8–10 weeks old and 22–25 g) were purchased from the Experimental Animal Laboratories of the Academy of Military Medical Sciences (Beijing, China). Mice were housed in animal facilities with 12-h light/dark cycle, controlled temperature and humidity, and free access to food and water. All procedures were approved by the Ethics Committee of Xuanwu Hospital, Capital Medical University, and were conducted in strict accordance with the ARRIVE Guidelines. Efforts were made to minimize the number of mice used and their suffering. In all experiments, mice were randomly assigned to individual groups; data were obtained by investigators blinded to the experimental design.

The procedures to establish the tMCAO/R model have been previously described [23]. Briefly, mice were anesthetized using isoflurane and were placed on a temperature-controlled stereotaxic frame (RWD Life Science, Shenzhen, China). The left common carotid artery (CCA), left external carotid artery (ECA), and left ICA were surgically exposed through a midline neck incision. A 6–0 monofilament nylon suture coated with silicon was inserted into the ICA through the ECA and slowly advanced to the MCA. Cerebral blood flow (CBF) was monitored using a non-invasive laser speckle imager (PeriCam PSI System, Perimed, Sweden), and successful MCAO was defined as regional CBF decreasing by > 80% from baseline. After 60 min of proximal MCA occlusion, the suture was removed to restore blood flow (reperfusion). Mice were then placed in heated cages to recover from anesthesia. Sham mice were anesthetized and received the same operating procedures without a suture being inserted into the MCA. All procedures were conducted with strict aseptic technique.

The following 3 experiments were conducted independently: (1) ANXA1 in the peri-infarct cortex and serum collected at 6, 12, 24, and 72 h post-tMCAO/R (*n* = 6 per time point) was quantified using western blotting and ELISA, respectively. A total of 60 mice were randomly assigned to the sham (*n* = 6) and tMCAO/R groups. (2) We studied the effects of Ac2-26 on the activated microglia/macrophage-mediated inflammatory brain damage after tMCAO/R. In total, 48 mice were randomly assigned into 2 groups of the tMCAO/R mice receiving intravenous (i.v.) infusion of either Ac2-26 (4 mg/kg/day; Cat. 1845, Tocris Bioscience, Bristol, UK) or the equal volume of sterile saline (vehicle) with the first dose at start of reperfusion. This dosage of Ac2-26 was selected according to previously published studies [20, 24] and our preliminary experiments. Post-treatment assessments were performed at 3 days post-tMCAO/R (Fig. 2a). (3) We investigated the role of FPR2/ALX receptor on the actions of Ac2-26 using an FPR2/ALX-specific antagonist Trp-Arg-Trp-Trp-Trp-Trp-NH2 (WRW4; Cat. 2262, Tocris Bioscience). We randomized 72 tMCAO/R mice to receive Ac2-26, Ac2-26 + WRW4, or vehicle. WRW4 (2.2 mg/kg; i.v.) were administered simultaneously with Ac2-26 at the start of reperfusion [20, 24]. Post-assessments were performed at 1 day (Fig. S1A) and 3 days post-tMCAO/R (Fig. 2a).

### Cell culture, OGD/R model, experimental design, and drug administration

The mouse BV2 microglial cells (American Type Culture Collection, Manassas, VA, USA) and mouse HT22 hippocampal neurons (China Infrastructure of Cell Line Resources, Beijing, China) were cultured in Dulbecco’s modified Eagle’s medium (DMEM, Corning, Tewksbury, MA, USA) supplemented with 10% fetal bovine serum (Sigma-Aldrich, MO, USA) and 1% penicillin–streptomycin (Hyclone, Logan, UT, USA). They were incubated at 37 °C in a humidified 5% carbon dioxide atmosphere, with medium changed every 2 days. To mimic cerebral I/R injury *in vitro*, cells were exposed to OGD/R as previously reported [25]. Briefly, the medium was replaced with D-glucose-free DMEM (Corning), and incubated at 37 °C in a hypoxic incubator (94% nitrogen and 5% carbon dioxide) for 2 h to simulate OGD damage. The cells were then transferred to normal glucose-containing DMEM medium in a normal incubator for an additional 22 h (reoxygenation). The following 2 experiments were conducted: (1) To evaluate the effects of Ac2-26 on microglia polarization and its mechanisms, Ac2-26 (1 or 5 μΜ; Tocris Bioscience) was added in the culture medium in the presence or absence of WRW4 (10μΜ; Tocris Bioscience) at the start of reoxygenation; the treated BV2 cells and conditioned medium (CM) were collected after 22 h of reoxygenation for further experiments (Fig. 6a). (2) We further investigated the effect of Ac2-26-mediated microglial polarization on post-I/R neuronal apoptosis using previously published methods [26, 27]. HT22 cells were subjected to OGD injury for 2 h, and then incubated with CM collected from OGD/R-stimulated BV2 cells with different treatments (collected from experiment 1) at the start of reoxygenation (Fig. 7a).

### Blood sample, brain tissue, and cell preparation

The sodium citrate-anticoagulated (0.36% of final concentration) blood samples were drawn from the forearms of healthy subjects and AIS patients, and the angular vein of sham and tMCAO/R mice. All blood samples were centrifuged at 1500 *g* for 15 min at room temperature (RT) to collect the supernatant, which were centrifuged at 13,000 *g* for 3 min at RT to collect cell-free plasma for ELISA. For nissl or immunofluorescence staining, anesthetized mice were sacrificed by transcardiac perfusion with ice-cold phosphate-buffered saline (PBS). The brains were dissected and fixed with 4% paraformaldehyde (PFA) for 24 h at RT, dehydrated in 20% and then 30% phosphate-buffered sucrose solutions, embedded in O.C.T. medium (Tissue-Tek, Torrance, CA), and sectioned. The peri-infarct cortex from tMCAO/R mice (Fig. 2a) and the same area from sham mice were dissected and frozen in liquid nitrogen until used for ELISA, western blotting, and quantitative real-time polymerase chain reaction (qRT-PCR). For ELISA, brain samples were homogenized in the lysis buffer on a rotary shaker for 90 min on ice, centrifuged at 1500 rpm for 15 min at 4 °C to collect the supernatant. In addition, the conditioned medium of BV2 cell cultures were harvested, centrifuged at 12,000 rpm for 10 min to remove debris, and the supernatants were collected for ELISA. For western blotting, proteins from brain samples or treated cells were extracted using a commercial protein extraction kit (Beyotime Biotech, Jiangsu, China). Total protein concentration was determined using a Nanodrop Spectrophotometer (ND-2000; ThermoFisher, Carlsbad, CA) at an optical density (OD) 280 nm. Total RNA for qRT-PCR were extracted from brain samples or BV2 cells using the TRIzol reagent (Invitrogen, Carlsbad, CA, USA) according to the manufacturer’s instructions. Spectrophotometric analysis at OD 260/280 nm (> 1.8) was used to ensure the purity and quantity of RNA.

### Neurobehavioral training and evaluation

A panel of behavioral tests was used to assess neurological function of mice at 3 days post-tMCAO/R.

Modified neurological severity score (mNSS) test, a composite test of motor, sensory, reflex, and balance functional of mice, was performed to evaluate the overall neurological deficits [28]. The score was graded from 0 to 18, where 0 represented normal performance, and 18 represented the most serious deficits. Sensorimotor and postural asymmetries were evaluated by corner test [29, 30]. Briefly, mice were placed between two plastic boards positioned at a 30° angle facing to the corner. When a mouse entered deep into the corner, vibrissae stimulation was applied to direct the mice to rear and subsequent turn to either side. Intact mice turned either side at equal frequencies whereas mice subjected to tMCAO/R would preferentially turn to the ipsilateral direction. The turns were recorded for 10 trials to calculate the mean percentage of ipsilateral turns. The foot fault test was performed to assess the sensorimotor coordination of the forelimbs as previously described [29, 30]. Briefly, a mouse was placed on a horizontal steel grid with different-sized openings for 1 min. Foot faults were recorded when the mouse inaccurately placed forelimbs, fell, or slipped between the wires. The foot faults of contralateral forelimbs were recorded for 3 trials to calculate the mean percentage of foot faults. The adhesive-removal test was performed to estimate tactile responses and asymmetries of an injured mouse [29, 30]. Briefly, two pieces of adhesive-backed paper dots (~ 50 mm^2^) were attached to the distal–radial region on the wrist of each forelimb as bilateral tactile stimuli. Before tMCAO/R induction, all mice were trained for 3 consecutive days for removing the dots within 20 s. On the testing day, the mean time (3 trials) taken by a mouse to remove the dots from the forelimbs was recorded with a maximal testing time of 5 min.

### ELISA

Levels of ANXA1 in cell-free plasma of human (Cat. ab222868) or mice (Cat. ab264613), and IL-1β (Cat. ab100705) and IL-10 (Cat. ab108870) in the peri-infarct cortex of tMCAO/R mice and culture supernatants of treated BV2 cell were detected and quantified by specific ELISA kits (all from Abcam, Cambridge, UK) following the manufacturer’s instructions.

### Nissl staining and infarct volume measurement

Nissl staining was used to measure volume of infarct [26]. Briefly, the coronal cryosections of the brain (20-μm thick, 1-mm intervals) were stained with cresyl violet (Cat. G1430; Solarbio, Beijing, China) for 10 min at 60 °C. Images were captured and analyzed using National Institute of Health (NIH) ImageJ software (Version 1.46r; Bethesda, MD, USA). The infarct area was calculated as non-infarcted contralateral hemisphere area minus surviving area of infarcted ipsilateral hemisphere. Infarct (V1) and contralateral hemisphere (V2) volumes were computed by numeric integration of sequential regions, and the results were presented as: Infarct volume (%) = V1/V2 × 100%.

### Cortical CBF

Cortical CBF was measured at baseline, 0 h (onset of MCAO), 1 h (onset of reperfusion), and 72 h post-tMACO/R using a noninvasive laser speckle imager with a 70-mW built-in laser diode and a 1388 × 1038-pixel charge-coupled device (CCD) camera (Perimed) as previously described [31]. Briefly, a mouse was anesthetized with isoflurane, positioned in the temperature-controlled stereotaxic frame (RWD Life Science), and a midline incision was made to expose the skull. The CCD camera was installed 10 cm above the skull, and CBF images were captured within a 2.0-cm × 2.0-cm scanning area. The ipsilateral cortical blood flow (perfusion unit, PU) was calculated using the vendor supplied PIMSoft software (version 1.5; Perimed) and presented as mean perfusion values.

### Immunofluorescence

Brain cryosections or treated cells (cultured on coverslips) were fixed for 20 min in precooled 4% PFA, and permeabilized with 0.1% Triton X-100 (Sigma-Aldrich) for 10 min at RT [28]. The fixed tissues and cells were blocked with 3% bovine serum albumin (Sigma-Aldrich) for 30 min at 37 °C and incubated with rabbit anti-ionized calcium-binding adapter molecule-1 (Iba-1; Cat. 019-19741, Wako, Osaka, Japan), rat anti-CD16/32 (Cat. 553142; BD Pharmingen, Franklin Lakes, NJ, USA), goat anti-CD206 (Cat. AF2535; R&D Systems, Minneapolis, MN, USA), rabbit anti-occludin (Cat. 40-4700, Invitrogen), rabbit anti-claudin-5 (Cat. 35-2500, Invitrogen), mouse anti-Iba-1 (Cat. MA5-27726, Invitrogen), rat anti-iNOS (Cat. 14-5920-82, Invitrogen), or rabbit Arg-1 (Cat. 93668, CST, Danvers, MA, USA) antibodies overnight at 4 °C. After rinsing with PBS, the tissues and cells were stained with corresponding secondary antibodies in the dark for 1 h at RT. Nuclei were counterstained with 4’,6-diamidino-2-phenyl-indole (DAPI; Abcam). Immunofluorescent double staining of neuronal nuclei (NeuN) and terminal deoxynucleotidyl transferase-mediated dUTP nick end labelling (TUNEL) was performed to quantify neuronal apoptosis. The fixed brain tissues were blocked and incubated with rabbit anti-NeuN antibody (Cat. ab177487, Abcam) at 4 °C overnight and subsequently subjected to TUNEL staining using an in Situ Cell Death Detection kit (Roche, Mannheim, Germany). In addition, TUNEL/DAPI staining was also performed to detect neuronal apoptosis in fixed HT22 cells according to the manufacturer’s instructions (Roche). Images were captured under a fluorescence microscope (Olympus IX81, Tokyo, Japan). Positive cells from 15 randomly selected microscopic fields (5 fields/section × 3 sections/mouse) were quantified using NIH ImageJ software.

### Western blotting

Western blotting was performed, as previously described [28]. Briefly, equal protein (10 μg per lane) was separated by sodium dodecyl sulfate-polyacrylamide gel electrophoresis, and transferred to polyvinylidene difluoride membranes (Millipore, Temecula, CA, USA). The membranes were blocked with 5% non-fat dried milk in Tris-buffered saline containing Tween-20 (TBST) at RT for 2 h and then incubated overnight at 4 °C with rabbit antibodies against ANXA1 (Cat. PA5-27315, Invitrogen), Iba-1 (Cat. ab178846, Abcam), CD16 (Cat. MA5-36143, Invitrogen), iNOS (Cat. ab178945, Abcam), CD206 (Cat. ab64693, Abcam), Arg-1 (Cat. 93668, CST), albumin (Cat. ab207327, Abcam), occludin (Cat. 40-4700, Invitrogen), claudin-5 (Cat. 35-2500, Invitrogen), adenosine 5’-monophosphate-activated protein kinase α subunit (AMPKα; Cat. 2532, CST), phosphor (p)-AMPKα (Thr172; Cat. 2531, CST), mammalian target of rapamycin (mTOR; Cat. 2972, CST), p-mTOR (Ser2448; Cat. 2971, CST), cleaved caspase-3 (Cat. 9664, CST), β-actin (Cat. ab8227, Abcam), or GAPDH (Cat. 2118, CST). Thereafter, membranes were washed with TBST, clipped according to the pre-stained protein ladder (Cat. 26620, Invitrogen), and then incubated with appropriate horseradish-peroxidase-conjugated goat anti-rabbit IgG (Cat. ZB-2305, Zsgb-bio, Beijing, China). The blot bands were observed with an enhanced chemiluminescence kit (Millipore) using a Gel Doc Imager (Bio-Rad, Hercules, CA, USA), and their intensities were analyzed using densitometry.

### qRT-PCR

qRT-PCR was performed, as previously described [28]. In brief, RNA was reverse-transcribed into cDNA with a SuperScript® III CellsDirect™ cDNA Synthesis Kit (Invitrogen). qRT-PCR was performed on an Opticon 2 Real-Time PCR Detection System (Bio-Rad) with the corresponding primers and SYBR Green PCR Master Mix (Applied Biosystems, Waltham, MA, USA). *GAPDH* was used as an internal control. The mRNA levels of target genes were normalized to that of *GAPDH* using the 2^−ΔΔCt^ method and were presented as relative expression. The primer sequences were as follows:

CD16: forward 5’-TTTGGACACCCAGATGTTTCAG-3’,

reverse 5′-GTCTTCCTTGAGCACCTGGATC-3’;

CD32: forward 5’-AATCCTGCCGTTCCTACTGATC-3’,

reverse 5’-GTGTCACCGTGTCTTCCTTGAG-3’;

iNOS: forward 5’-GGTGAAGGGACTGAGCTGTT-3’,

reverse 5’-ACGTTCGTTCTCTTGCA-3’;

Arg-1: forward 5’-CACCTGAGCTTTGATGTCG-3’,

reverse 5’-TGAAAGGAGCCCTGTCTTG-3’;

YM1: forward 5’-GAGGTAATGAGTGGGTTGG-3’,

reverse 5’-ACGGCACCTCCTAAATTGT-3’;

CD206: forward 5’-AAGGAAGGTTGGCATTTGT-3’,

reverse 5’-CTTTCAGTCCTTTGCAAGC-3’;

GAPDH: forward 5’-GCCAAGGCTGTGGGCAAGGT-3’,

reverse 5’-TCTCCAGGCGGCACGCAGA-3’.

### Evans blue (EB) dye extravasation

EB solution (2% given at 4 mL/kg, dissolved in PBS; Sigma-Aldrich) was infused into mice through the tail vein and allowed to circulate for 2 h, as previously described [28]. The mice were euthanized and transcardially perfused with ice-cold PBS to remove the intravascular EB dye. For fluorescence images, brain cryosections were sectioned, fixed in precooled 4% PFA, stained with DAPI, and observed under an IX81 fluorescence microscope (Olympus). For quantitative analysis, the brains were harvested, weighed, homogenized in formamide (1:20 w/v), and incubated at 60 °C for 72 h. The brain homogenate was centrifuged at 14,000 rpm for 30 min to collect the supernatant, in which EB was determined at OD 620 nm using a SpectraMax M5 plate-reader (Molecular Devices, Sunnyvale, CA), quantified using a linear standard, and expressed as μg/g brain tissue.

### Lactate dehydrogenase (LDH) cytotoxicity assay

The cell death of treated HT22 cells was assessed by measuring levels of LDH in the culture supernatants using a LDH cytotoxicity assay kit (Cat. BC0685, Solarbio) according to the manufacturer’s instructions. The absorbance at OD 450 nm was measured using a SpectraMax M5 plate-reader (Molecular Devices).

### Statistical analysis

All statistical analyses were performed with SPSS 22.0 statistical software (IBM, Armonk, NY, USA). Preliminary analysis of data normality was performed with Shapiro-Wilk’s test. Plasma ANXA1 obtained from AIS patients and healthy controls did not match normality and were presented as median and interquartile range (IQR). Differences between groups were analyzed using Mann–Whitney U test. Spearman correlation coefficient was used to assess the correlation between plasma ANXA1 and 3-month mRS scores. All other continuous variables were normally distributed, and were presented as mean ± standard deviation (SD). Categorical variables were presented as frequency and percentage. Differences between 2 groups were assessed using the independent-samples t test for continuous measures, and the chi-square test for categorical variables (or Fisher’s exact test when the expected value was < 5). When comparing 3 or more groups, one-way analysis of variance (ANOVA) followed by post hoc Bonferroni's multiple comparison test was performed. A *p* < 0.05 was considered statistically significant.

## Results

### Plasma levels of ANXA1 in AIS patients before and after EVT

**Fig. 1:**
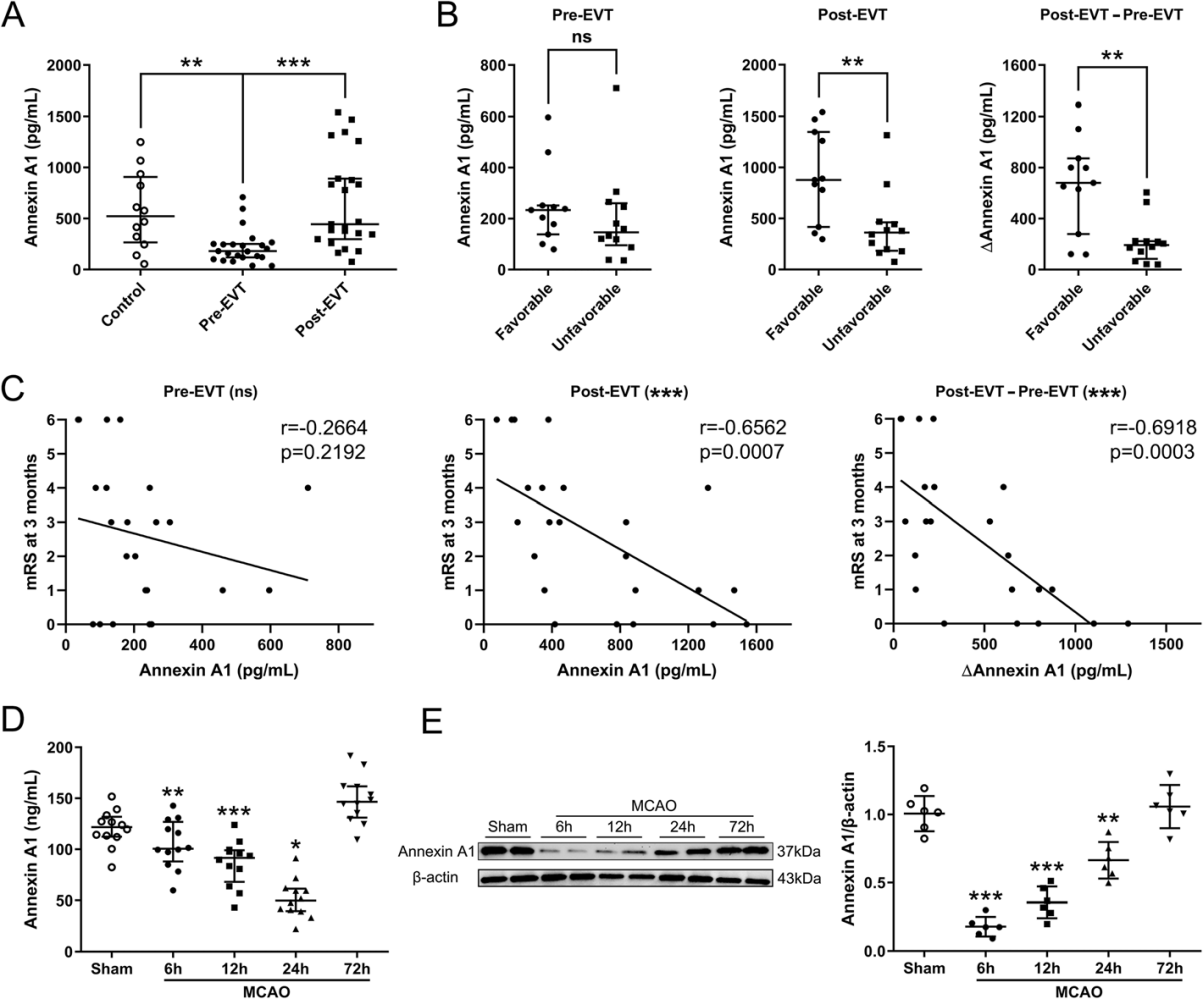
Expression profiles of endogenous ANXA1 in EVT-treated AIS patients and tMCAO/R-injured mice. **a** Plasma ANXA1 levels detected by ELISA in normal control subjects (*n* = 12) and AIS patients treated with EVT (*n* = 23). Data were presented as median and IQR, and were analyzed by Mann–Whitney U test. **p < 0.01, and ***p < 0.001. **b** Plasma ANXA1 levels or ∆ANXA1 levels in EVT-treated AIS patients with favorable (*n* = 11) and unfavorable (*n* = 12) clinical outcomes. ∆ANXA1: ANXA1 concentration on 2–3 days post-EVT minus pre-EVT ANXA1 concentration. Data were presented as median and IQR and were analyzed by Mann–Whitney U test. *ns*, not significant. **p < 0.01. **c** Spearman correlation coefficient analyses of correlation between plasma ANXA1 levels or ∆ANXA1 levels in AIS patients and 3-month mRS scores. **d** ELISA analyses of the expressions of ANXA1 in peripheral blood of tMCAO/R-injured mice. Data were presented as the mean ± SD (*n* = 12/group), and were analyzed by one-way ANOVA followed by Bonferroni’s multiple comparison test. *p < 0.05, **p < 0.01, and ***p < 0.001. **e** Representative western blotting bands and densitometric quantifications of ANXA1 in the peri-infarct cortex after tMCAO/R. Data were presented as the mean ± SD (*n* = 6/group) and were analyzed by one-way ANOVA followed by Bonferroni’s multiple comparison test. **p < 0.01, and ***p < 0.001

Twenty-three AIS patients who achieved successful recanalization by EVT were included in this study (68.96 ± 9.76 years old, 56.52% of male). The patients and the controls’ demographic and clinical characteristics were summarized in Supplemental Table 1. Of the 23 patients, 11 (47.83%) had favorable prognosis (favorable group) and 12 (52.17%) had unfavorable prognosis (unfavorable group) at 3 months post-EVT. Baseline characteristics were comparable between AIS patients and healthy controls and between patients in the favorable group and those in the unfavorable group, except for the frequency rate of atrial fibrillation. Plasma levels of ANXA1 were significantly lower in AIS patients measured at admission than those of healthy controls (Fig. 1a). They were significantly increased on 2–3 days post-EVT to the levels comparable to those of control subjects (Fig. 1a). Subgroup analysis revealed that while ANXA1 was similar between patients in the favorable and those in the unfavorable group before EVT (left panel, Fig. 1b), it was markedly higher on 2–3 days post-EVT in patients of the favorable group (middle panel, Fig. 1b). In addition, the degree of ANXA1 elevation (∆ANXA1, defined as ANXA1 concentration on 2–3 days post-EVT minus pre-EVT ANXA1 concentration) was also significantly higher in the favorable group (right panel, Fig. 1b). Furthermore, the increased ANXA1 levels on 2–3 days post-EVT as well as ∆ANXA1 levels were inversely correlated with mRS scores at 3 months post-EVT (Fig. 1c). These results indicated that ANXA1 may predict EVT prognosis and guide reperfusion therapies.

### Levels of ANXA1 in the peri-infarct cortex and peripheral blood after tMCAO/R

Consistent with the findings in patients, ANXA1 was gradually decreased in the plasma of tMCAO/R mice, reaching the lowest point at 24 h, followed by a significant increase to higher levels than that of sham mice at 72 h (Fig. 1d). The reduction of ANXA1 expression was also detected in the peri-infarct cortex, with the lowest level at 6 h, followed by gradual increase therefore after, reaching the control level (sham) at 72 h (Fig. 1e). These results demonstrated that mice subjected to tMCAO/R produced the dynamic changes of plasma ANXA1, suggesting that reduced endogenous ANXA1 might contribute to the cerebral/ I/R injury, and upregulation of ANXA1 might therefore have adjunct therapeutic benefits.

### Ac2-26 ameliorated brain injury after tMCAO/R

**Fig. 2:**
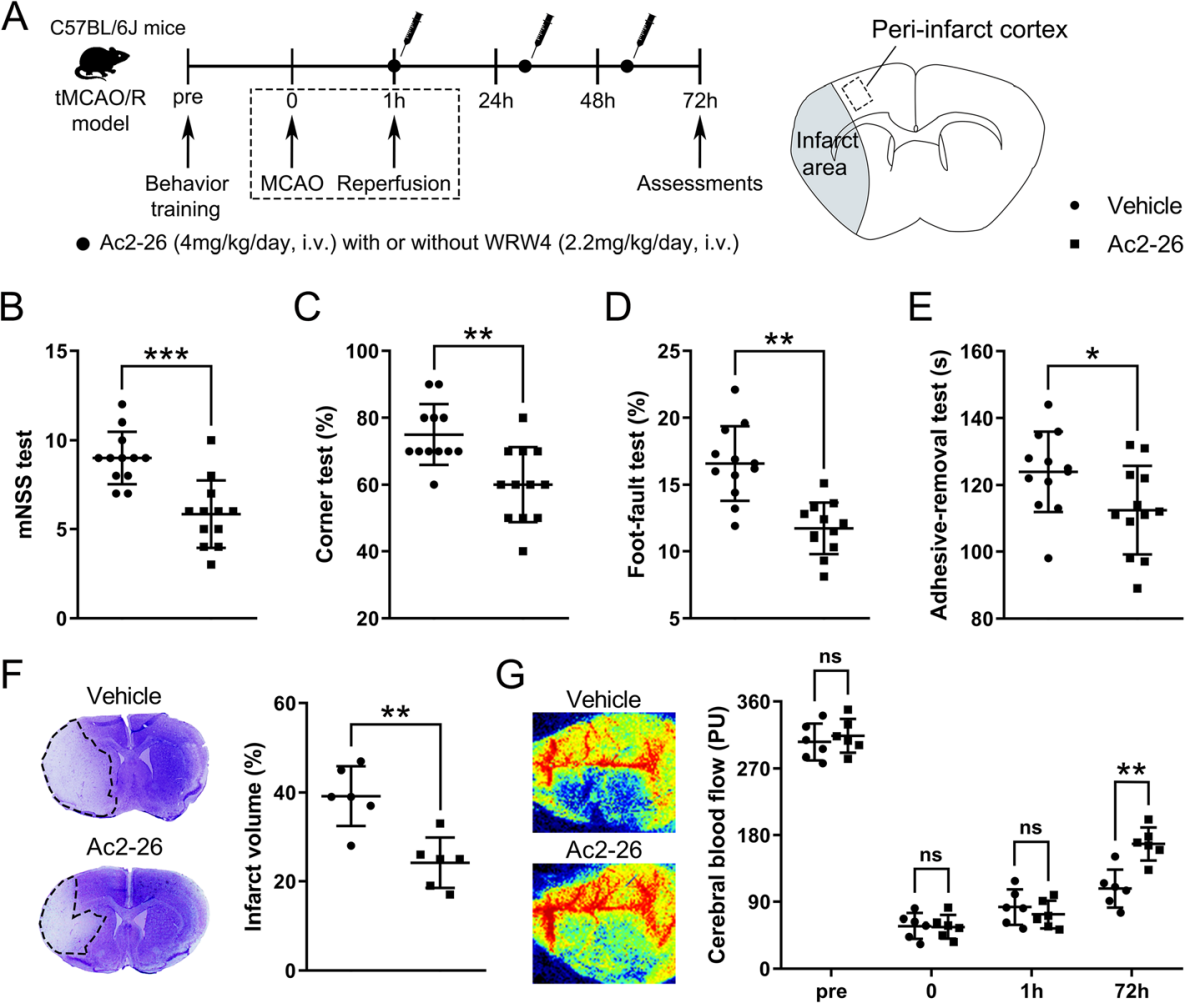
Effect of Ac2-26 on neurological function, cerebral infarct volume, and cortical CBF at 3 days post-tMCAO/R. **a** Schematic diagram of the experimental design. **b–e** Neurological function was evaluated by mNSS test (**b**), corner test (**c**), foot fault test (**d**), and adhesive removal test (**e**). **f** Representative photographs of nissl staining and quantitative analyses of cerebral infarct volume. **g** Representative photographs of laser speckle contrast imaging and quantitative analyses of cortical CBF. *PU*, perfusion unit. Data were presented as the mean ± SD (**b**–**e** *n* = 12/group; **f**–**g** *n* = 6/group) and were analyzed by independent samples t test. *p < 0.05, **p < 0.01, and ***p < 0.001

Ac2-26 mimics the pharmacophore of ANXA1 (N-terminal proteolytic cleavage products) and is reported to exert similar biological effects as the full ANXA1 [14]. We therefore utilized Ac2-26 to explore its therapeutic effects on cerebral I/R injury. Ac2-26 significantly improved the neurological function of tMCAO/R mice, as measured by mNSS (Fig. 2b and Fig. S1B), corner test (Fig. 2c), foot fault test (Fig. 2d), and adhesive removal test (Fig. 2e). Ac2-26 treatment significantly reduced the volume of cerebral infarct (Fig. 2f) and increased cortical CBF (Fig. 2g), as compared with mice receiving vehicle buffer. These results demonstrated that Ac2-26 effectively ameliorated I/R injury.

### Ac2-26 inhibited M1 polarization and promoted M2 polarization after tMCAO/R

**Fig. 3:**
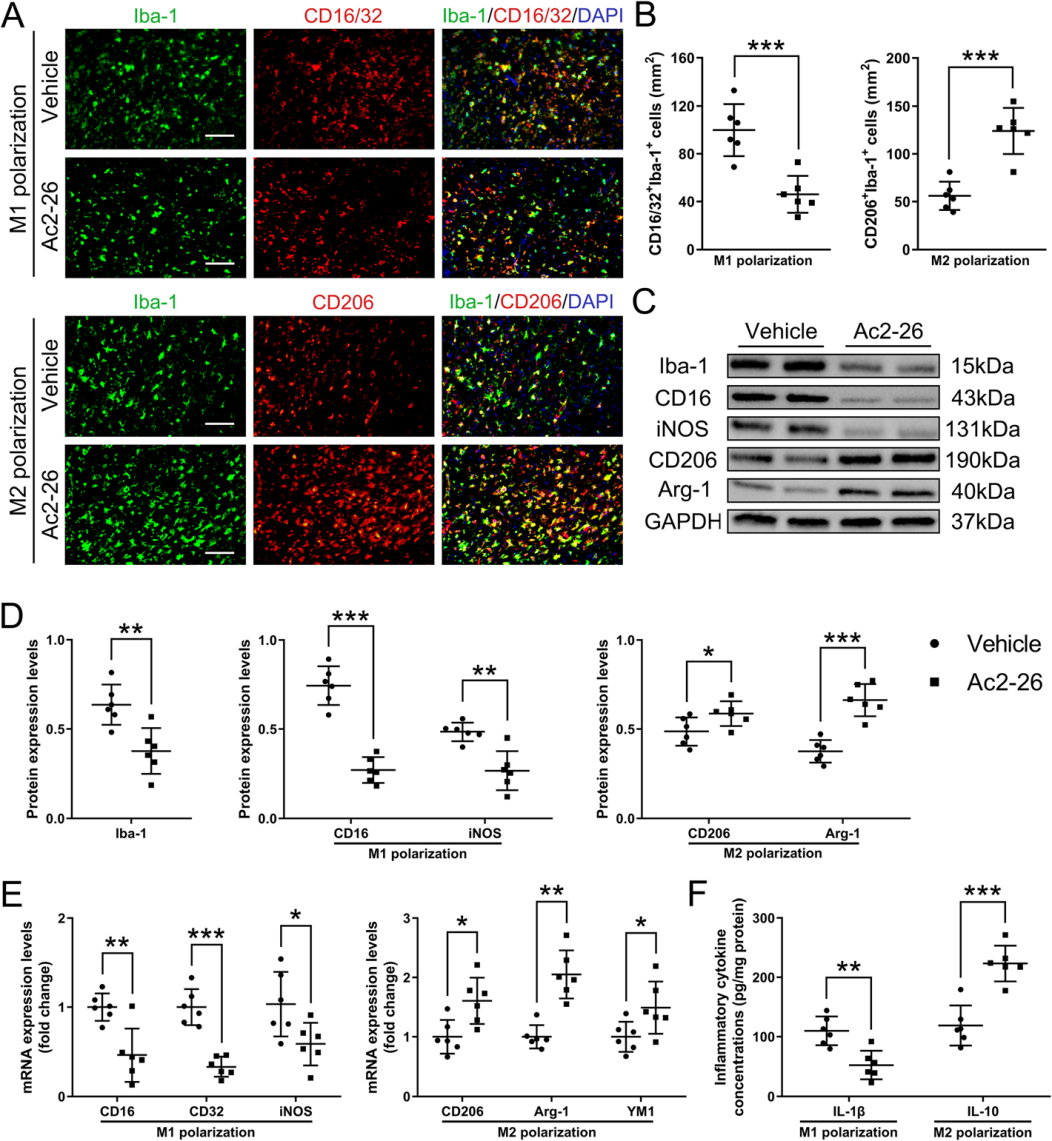
Effect of Ac2-26 on microglial/macrophage polarization in the peri-infarct cortex at 3 days post-tMCAO/R. **a–b** Representative photographs of double immunostaining and quantitative analyses of microglial/macrophage polarization. M1-phenotype: CD16/32^+^ (red) and Iba-1^+^ (green); M2-phenotype: CD206^+^ (red) and Iba-1^+^ (green). Scale bar = 200 μm. **c–d** Representative western blotting bands and densitometric quantifications of activated microglial/macrophage marker Iba-1, M1-phenotype markers (CD16 and iNOS), and M2-phenotype markers (CD206 and Arg-1). **e** qRT-PCR analyses of mRNA expressions of M1-phenotype markers (CD16, CD32, and iNOS) and M2-phenotype markers (CD206, Arg-1, and YM1). **f** ELISA analyses of the expressions of a pro-inflammatory cytokine IL-1β (M1-phenotype) and an anti-inflammatory cytokine IL-10 (M2-phenotype). Data were presented as the mean ± SD (*n* = 6/group) and were analyzed by independent samples t test. *p < 0.05, **p < 0.01, and ***p < 0.001

To further define the pathway through which Ac2-26 improved outcomes, we detected the state of microglial/macrophage polarization using surface markers specific for M1 and M2 and also profiled the cytokine expression associated with the polarization. Ac2-26 treatment significantly reduced the number of activated microglia/macrophages (Iba-1^+^) that express the M1 marker CD16/32^+^ in the peri-infarct cortex at 3 days post-tMCAO/R as compared with those receiving the vehicle, while it increased the M2 marker CD206^+^ (Fig. 3a and b), suggesting an M1-to-M2 transition. The findings made with immunofluorescence microscopy were further validated using western blotting (Fig. 3c and d and Fig. S1D) and qRT-PCR (Fig. 3e and Fig. S1E) analyses at 1 and 3 days post-tMCAO/R, as the M1 markers CD16, CD32, and iNOS decreased and the M2 markers CD206, Arg-1, and YM1 increased. Ac2-26 also significantly suppressed the secretion of pro-inflammatory IL-1β and enhanced the secretion of anti-inflammatory IL-10 (Fig. 3f and Fig. S1F). In addition, the expression of Iba-1 decreased significantly in mice treated with Ac2-26 (Fig. 3c and d and Fig. S1D), indicating that Ac2-26 may have reduced microglia activation and macrophage invasion. These results demonstrated that Ac2-26 alleviated cerebral I/R injury by promoting the polarization of microglia/macrophages to M2 phenotype.

### Ac2-26 ameliorated BBB disruption and neuronal apoptosis after tMCAO/R

**Fig. 4:**
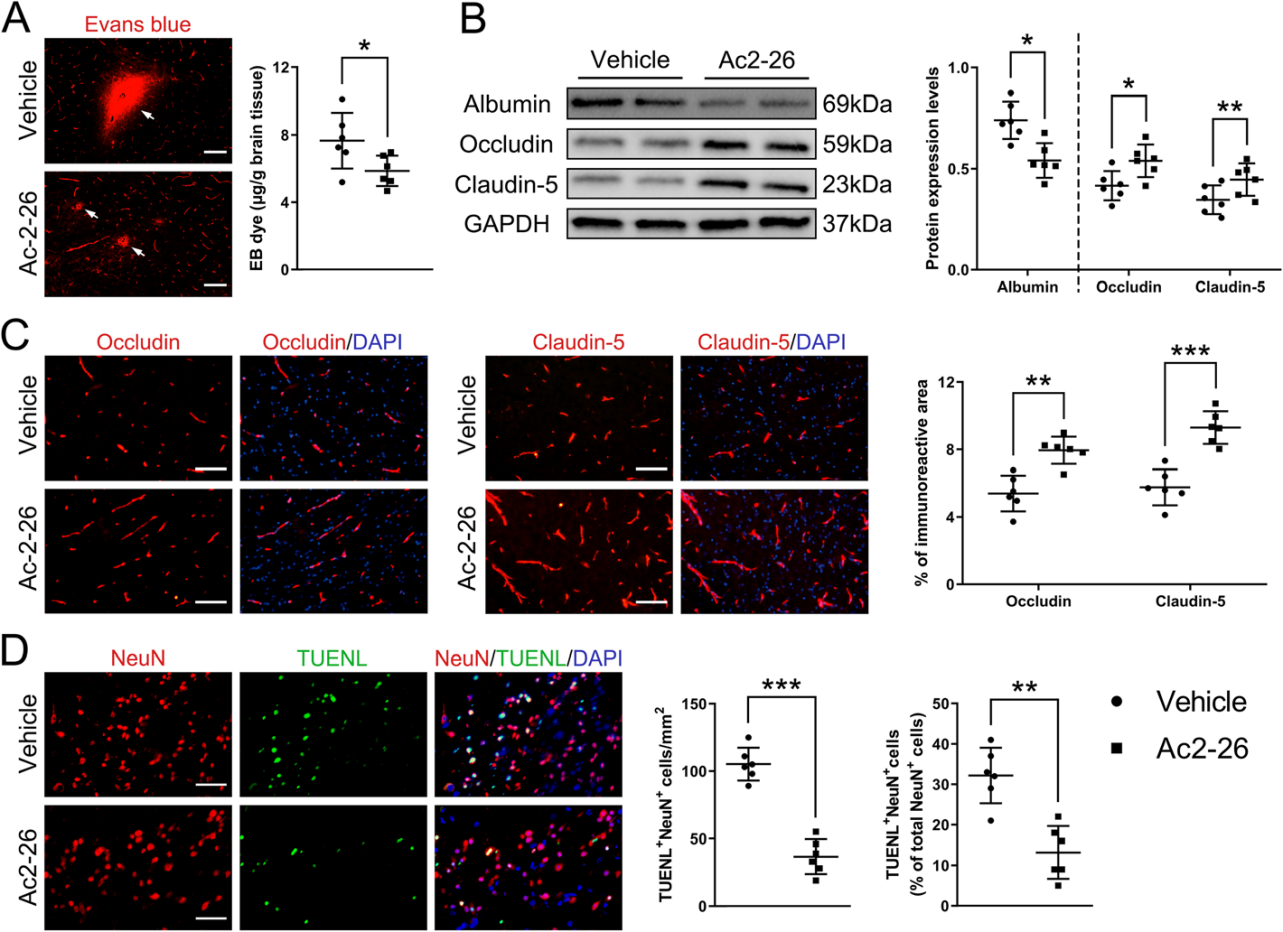
Effect of Ac2-26 on BBB disruption and neuronal apoptosis in the peri-infarct cortex at 3 days post-tMCAO/R. **a** Representative photographs of immunostaining and quantitative analyses of EB dye extravasation. White arrows indicated exudative EB dye. Scale bar = 100 μm. **b** Representative western blotting bands and densitometric quantifications of albumin extravasation and TJ (occludin and claudin-5) expressions. **c–d** Representative photographs of immunostaining and quantitative analyses of TJ (occludin and claudin-5; red) expression and neuronal apoptosis (NeuN, red; TUNEL, green). Scale bar = 200 μm. Data were presented as the mean ± SD (*n* = 6/group), and were analyzed by independent samples t test. *p < 0.05, **p < 0.01, and ***p < 0.001

Over-activated microglia/macrophages have been shown to exacerbate BBB disruption and neuronal apoptosis after tMCAO/R [12]. Mice treated with Ac2-26 had significantly reduced endothelial permeability induction measured by EB (Fig. 4a and Fig. S1C) and albumin (Fig. 4b) into the peri-infarct cortex. This improvement was consistent with enhanced expression of the tight junction proteins occludin and claudin-5 (Fig. 4b and c). The TUNEL and NeuN double immunostaining showed Ac2-26 decreased the number of apoptotic cells, primarily neurons, in the peri-infarct cortex (Fig. 4d). These results suggested that Ac2-26 could attenuate BBB damage and neuronal apoptosis, and these protective roles might partially be dependent on regulated microglia/macrophage polarization and reduced subsequent cerebral inflammatory responses.

### Ac2-26 regulated FPR2/ALX-dependent AMPK-mTOR pathway to control microglial/macrophage polarization both *in vivo* and *in vitro*

**Fig. 5:**
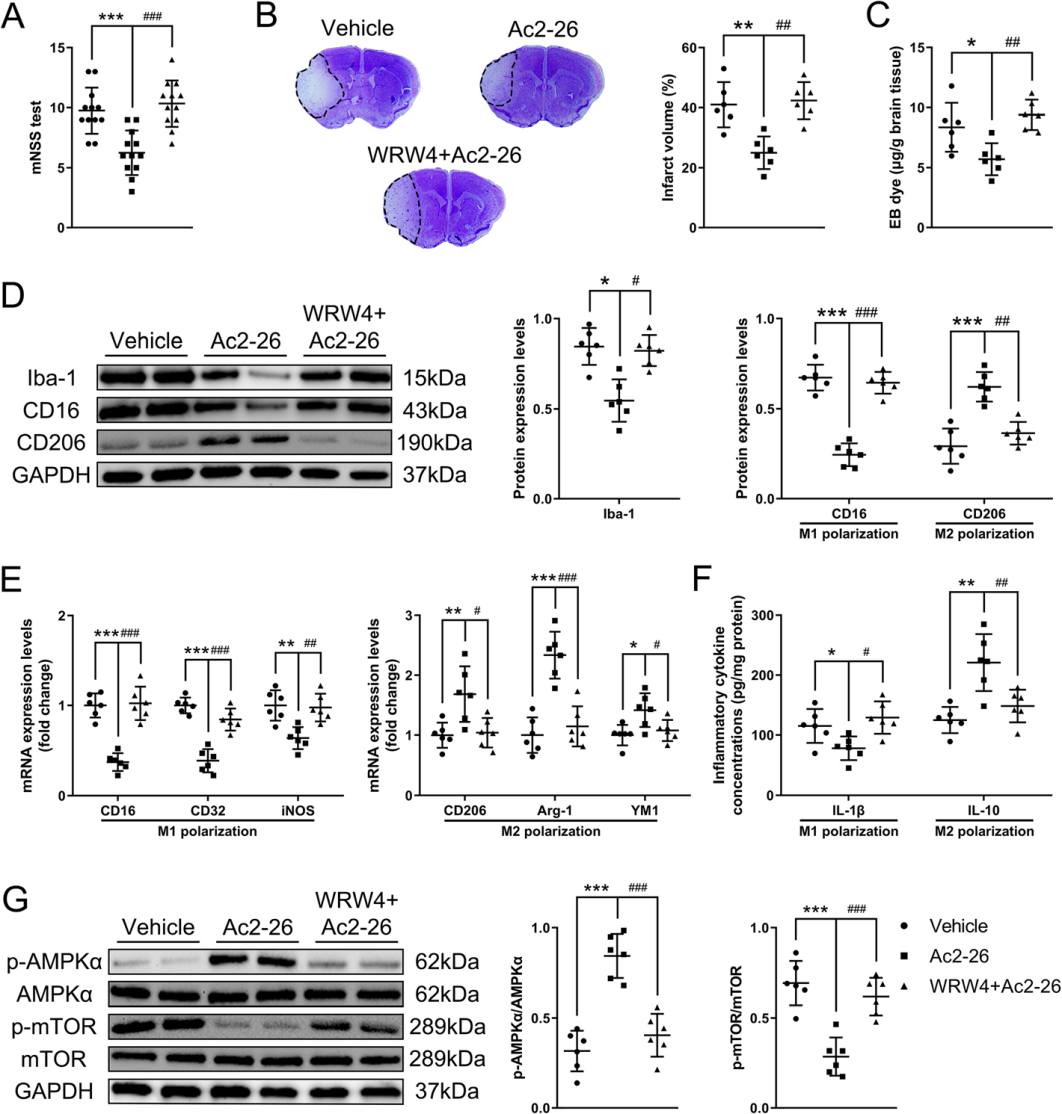
Effect of FPR2/ALX-dependent AMPK-mTOR pathway on Ac2-26-mediated microglial/macrophage polarization in the peri-infarct cortex at 3 days post-tMCAO/R. **a** Neurological function was evaluated by mNSS test. **b** Representative nissl staining and quantitative analyses of cerebral infarct volume. **c** Quantitative analyses of EB dye extravasation. **d** Representative western blotting bands and densitometric quantifications of activated microglial/macrophage marker Iba-1, M1-phenotype marker CD16, and M2-phenotype marker CD206. **e** qRT-PCR analyses of mRNA expressions of M1-phenotype markers (CD16, CD32, and iNOS) and M2-phenotype markers (CD206, Arg-1, and YM1). **f** ELISA analyses of the expressions of a pro-inflammatory cytokine IL-1β (M1-phenotype) and an anti-inflammatory cytokine IL-10 (M2-phenotype). **g** Representative western blotting bands and densitometric quantifications of phosphorylation of AMPKα and mTOR. Data were presented as the mean ± SD (**A** n = 12/group; **b**–**g** *n* = 6/group) and were analyzed by one-way ANOVA followed by Bonferroni’s multiple comparison test. *p < 0.05, **p < 0.01, and ***p < 0.001, ^#^*p* < 0.05, ^##^*p* < 0.01, and ^###^*p* < 0.001

**Fig. 6:**
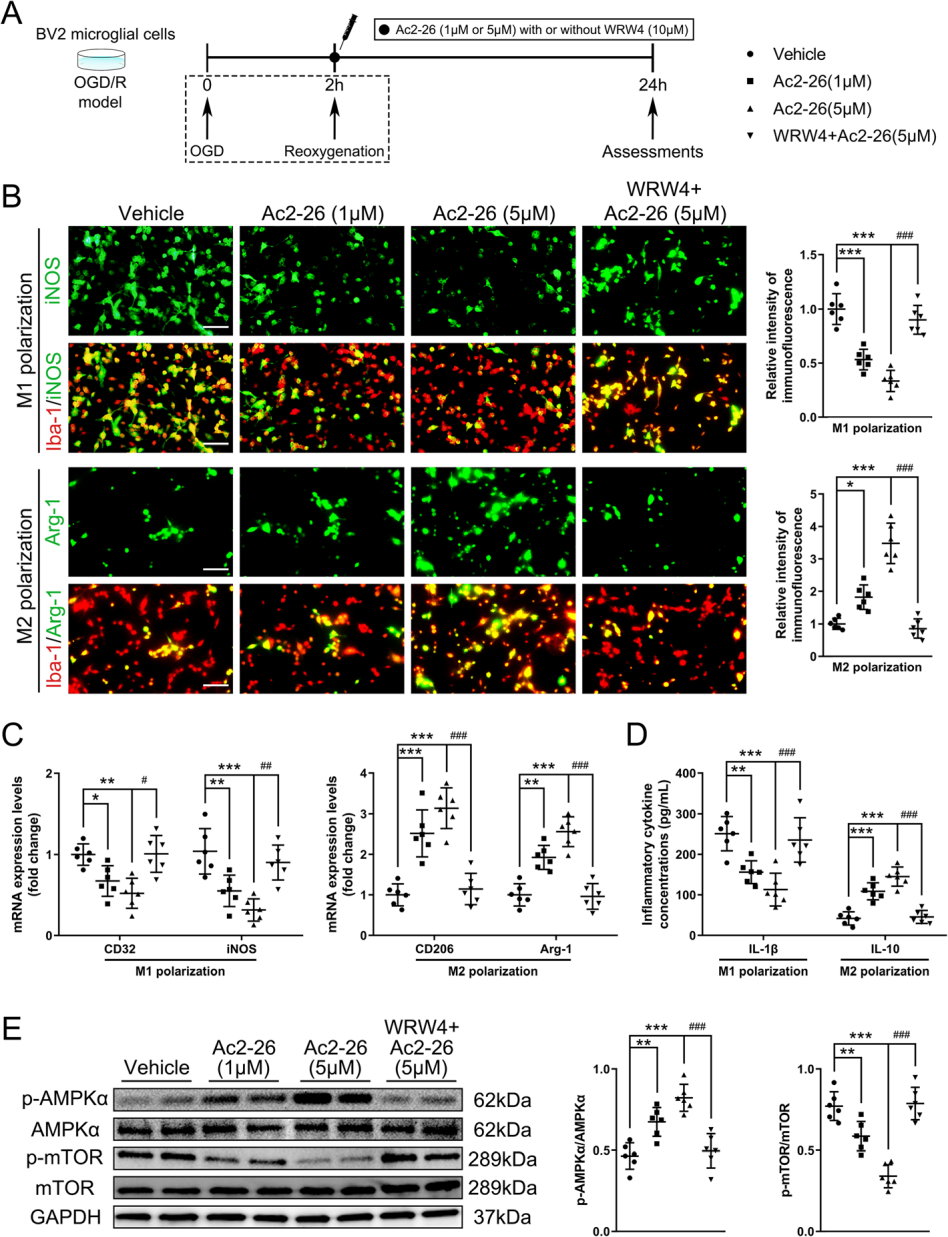
Effect of FPR2/ALX-dependent AMPK-mTOR pathway on Ac2-26-mediated *in vitro* BV2 microglial polarization. **a** Schematic diagram of the experimental design. **b** Representative photographs of double immunostaining and quantitative analyses of BV2 microglial polarization. M1-phenotype: iNOS^+^ (green) and Iba-1^+^ (red); M2-phenotype: Arg-1^+^ (green) and Iba-1^+^ (red). Scale bar = 100 μm. **c** qRT-PCR analyses of mRNA expressions of M1-phenotype markers (CD32 and iNOS) and M2-phenotype markers (CD206 and Arg-1). **d** ELISA analyses of the expressions of a pro-inflammatory cytokine IL-1β (M1-phenotype) and an anti-inflammatory cytokine IL-10 (M2-phenotype). **e** Representative western blotting bands and densitometric quantifications of phosphorylation of AMPKα and mTOR. Data were presented as the mean ± SD (*n* = 6/group) and were analyzed by one-way ANOVA followed by Bonferroni’s multiple comparison test. *p < 0.05, **p < 0.01, and ***p < 0.001, ^#^*p* < 0.05, ^##^*p* < 0.01, and ^###^*p* < 0.001

We next ascertained whether the regulatory effects of Ac2-26 were mediated by interacting with FPR2/ALX receptor and explored its downstream signaling pathway. The selective FPR2/ALX receptor antagonist WRW4 significantly abrogated the protective effects of Ac2-26 on neurological deficits (Fig. 5a and Fig. S1B), cerebral infarct (Fig. 5b), BBB disruption (Fig. 5c and Fig. S1C), microglia activation and macrophage invasion (Fig. 5d and Fig. S1D), and M2 polarization (Fig. 5d–f and Fig. S1D-F) at 1 day and 3 days post-tMCAO/R. Furthermore, Ac2-26 significantly promoted the phosphorylation of AMPKα and inhibited the phosphorylation of mTOR; these actions were again blocked by WRW4 (Fig. 5g). To further define the involvement of FPR2/ALX-dependent AMPK-mTOR pathway in Ac2-26 protection without the confounding influences of mouse models, we conducted *in vitro* OGD/R experiments. In cultured BV2 cells, we first examined the effect of Ac2-26 on post-OGD/R cell viability, and found that Ac2-26 did not affect BV2 cell viability (Fig. S2). Ac2-26 reduced the intensity of iNOS^+^ Iba-1^+^ staining whereas enhanced the intensity of Arg-1^+^ Iba-1^+^ staining in a dose-dependent manner. WRW4 given together with Ac2-26 abrogated the effects (Fig. 6b). Similarly, mRNA levels in cell lysates and cytokine productions in supernatants of OGD/R-stimulated BV2 cells confirmed that Ac2-26 suppressed the M1 markers CD32, iNOS, and IL-1β and enhanced the M2 markers CD206, Arg1, and IL-10 in a dose-dependent manner, and WRW4 blocked this Ac2-26-induced polarization shift of microglia (Fig. 6c and d). Moreover, Ac2-26 enhanced the phosphorylation of the AMPKα and subsequent inhibition of mTOR in a dose-dependent manner, and both activities were reversed by WRW4 (Fig. 6e). These results confirmed that Ac2-26 modulated the polarization state of microglia/macrophage via FPR2/ALX-dependent AMPK-mTOR pathway.

### Ac2-26 restrained post-OGD/R neuronal apoptosis via modulating microglial/macrophage polarization

**Fig. 7:**
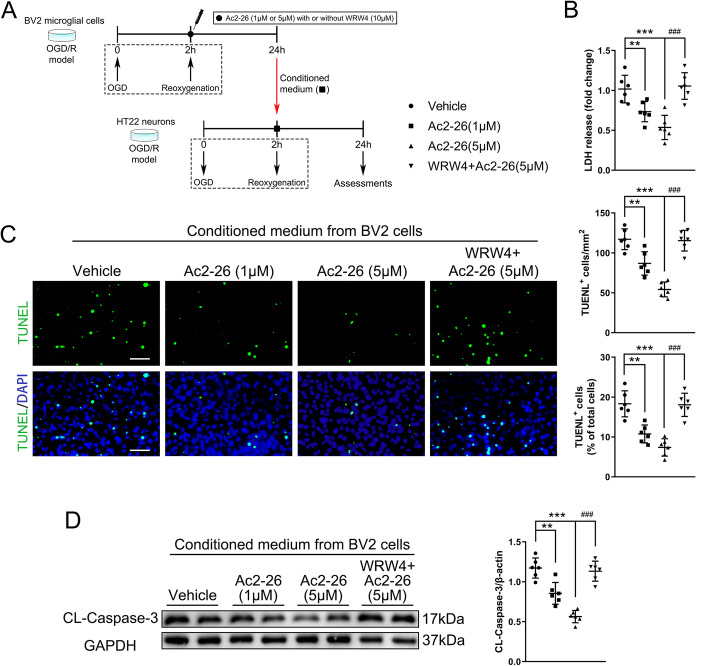
Effect of Ac2-26-mediated microglia polarization on post-OGD/R HT22 neuronal apoptosis. **a** Schematic diagram of the experimental design. **b** Quantitative analyses of treated HT22 neuronal death using LDH release assay. **c** Representative photographs of immunostaining and quantitative analyses of HT22 neuronal apoptosis (TUNEL, green). Scale bar = 100 μm. **d** Representative western blotting bands and densitometric quantifications of cleaved-caspase-3. CL: cleaved. Data were presented as the mean ± SD (*n* = 6/group) and were analyzed by one-way ANOVA followed by Bonferroni's multiple comparison test. **p < 0.01, and ***p < 0.001, ^###^*p* < 0.001

To further confirm that Ac2-26 could indirectly protect against post-I/R neuronal apoptosis through mediating microglial/macrophage polarization and the subsequent inflammatory responses, we incubated OGD-injured HT22 cells with CM collected from OGD/R-stimulated BV2 cells with different treatments at the start of reoxygenation. When compared with CM of OGD/R-stimulated BV2 cells treated with vehicle, post-OGD/R HT22 cells treated with CM collected from Ac2-26-treated BV2 cells showed significantly lower cell death and apoptosis as indicated by the decreased release of LDH (Fig. 7b), number of TUNEL^+^ apoptotic cells (Fig. 7c), and expression of cleaved (activated) caspase-3 (a classic marker of apoptotic cells; Fig. 7d). However, these effects were again significantly reversed when post-OGD/R HT22 cells cultured in CM of BV2 cells were treated with Ac2-26 together with WRW4. These results suggested that Ac2-26-mediated polarization of microglia/macrophages to M2 phenotype could modulate post-I/R microenvironment, thus indirectly protecting against post-I/R neuronal apoptosis.

## Discussion

The main findings of the present study were that (1) plasma ANXA1 was reduced during the onset of AIS and recovered after successful recanalization by EVT; (2) the recovery of plasma ANXA1 predicted favorable prognosis of AIS patients; (3) Ac2-26 administered at the start of reperfusion alleviated cerebral I/R injury in the mouse model by shifting microglia/macrophage polarization toward anti-inflammatory M2 phenotype, and the findings in mice were validated through *in vitro* experiments; and (4) Ac2-26 modulated microglia/macrophage polarization by binding the FPR2/ALX receptor to activate the AMPK-mTOR pathway.

Randomized and controlled trials have demonstrated that AIS patients treated with EVT had a higher rate of successful recanalization, more favorable clinical outcomes, and longer therapeutic window compared with thrombolytic therapy [2, 3]. However, futile recanalization, which is defined as patients having unfavorable outcomes (mRS of 3–6) despite successful recanalization (mTICI of 2b–3), remains a major clinical challenge to predict and is poorly understood [4, 5]. There is no reliable clinical, blood, or radiological markers to predict outcomes after EVT [3, 32], except for more generic measurements such as age, stroke severity, general anesthesia, and hyperglycemia (glycated hemoglobin > 6.5%) for predicting poor prognosis of patients after EVT [5, 32]. Biomarkers detected in the peripheral blood of patients could have better predictive values because they are often related to pathophysiological response to EVT recanalization. In this regard, matrix metalloprotease-9 [6] and low-density lipoprotein cholesterol [33] have been associated with clinical outcomes in EVT-treated AIS patients. Senchenkova et al. [20] have shown that plasma levels of ANXA1 were lower in AIS patients than healthy controls, but the study did not link the finding to clinical outcomes of the AIS patients and those with EVT. In the present study, we first retrospectively collected 23 AIS patients with successful recanalization by EVT and confirmed that the plasma levels of ANXA1 were markedly lower in AIS patients than in healthy controls. Furthermore, our main findings were that we also showed the full recovery of plasma ANXA1 after EVT to the levels found in healthy subjects and associated higher plasma ANXA1 or plasma ∆ ANXA1 with favorable outcomes at 3 months after EVT. Moreover, we also reproduced the temporary dynamics of circulating ANXA1 in the mouse model of tMCAO/R. While it is not known if the low ANXA1 persists before stroke and can be linked to underlying conditions for stroke such as atherosclerosis, this finding suggest that plasma ANXA1 may predict clinical outcomes of AIS and the effectiveness of EVT therapies. In addition, the cause of reduced plasma ANXA1 is not known; it can be consumptive because ANXA1 is a member of annexins that share the ability to bind the anionic phospholipid phosphatidylserine, which is expressed on the surface of injured and apoptotic cells that increase significantly during AIS [34]. Alternatively, the synthesis and release of ANXA1 may be inhibited during AIS. However, this is unlikely because the postmortem of infarcted brains found enhanced expression of ANXA1 in the peri-infarct regions [35]. Interestingly, we found that ANXA1 was reduced in the peri-infract cortex immediately after tMCAO/R, but recovered 3 days after the recanalization, suggesting defined time-dependent changes in ANXA1 expression. The acute and transient decrease of endogenous ANXA1 let us speculate that the protection of ANXA1 may be abrogated in some reason at the early stage of cerebral I/R injury, and upregulation of ANXA1 may therefore have adjunct therapeutic benefits for AIS patients who achieved successful recanalization. However, given the relatively small sample size and retrospective design, a larger prospective or randomized study will be needed to validate these clinical findings.

The cerebral I/R injury has been extensively demonstrated to contribute to the development of futile recanalization after EVT [7, 10], and involves the rapid activation and polarization of microglia/macrophages and the subsequent immune and inflammatory responses in the ischemic penumbra [11, 12]. The activated microglia/macrophages were recruited to the peri-infarct regions and dynamically polarize a transient anti-inflammatory and protective M2-phenotype (day 0–3 after reperfusion) that is reversed to a sustained pro-inflammatory and detrimental M1-phenotype (day 3–14 after reperfusion), creating a vulnerable tissue microenvironment at the acute stage of I/R injury [11, 13]. Numerous *in vivo* and *in vitro* experimental studies have revealed that inhibiting M1-phenotype and/or stimulating M2-phenotype in the ischemic penumbra during an early phase could protect against cerebral I/R injury [11]. ANXA1, mainly expressed and secreted by microglia and endothelial cells of the adult human and rodent brains, has been shown to reduce cerebral I/R-associated complications [14, 36]. Experimental studies demonstrated that endogenous or exogenous ANXA1 or Ac2-26 alleviated brain damage by inhibiting leukocyte recruitment and pro-inflammatory mediator production and promoting microglial/macrophage efferocytosis of apoptotic neutrophils and cellular debris in the acute phase of cerebral I/R injury [18, 19]. More recently, studies from Gavins laboratory demonstrated that ANXA1 also has anti-thrombotic capabilities as evidenced by reducing platelet activation, neutrophil–platelet aggregation, and microvascular thrombosis [20, 24]. In the present study, we expanded the notion that Ac2-26 remarkably reduced microglia/macrophage activation and shifted their polarization states toward the neuroprotective and tissue-reparative M2-phenotype in the ischemic penumbra, thus alleviating BBB disruption and neuronal apoptosis, and improving outcomes at 1 day and 3 days post-tMCAO/R. Similarly, Li et al. [37] recently demonstrated that SUMOylation of endogenous ANXA1 in microglia/macrophages contributed to phenotype shifts in the mouse model of tMCAO/R. Furthermore, our studies in OGD/R-stimulated BV2 cells and HT22 cells confirmed the direct and dose-dependent effects of Ac2-26 on microglial polarization and indirect effects of Ac2-26 on neuronal apoptosis. These results were consistent with previous studies in which ANXA1 reduced primary or BV2 microglial activation, migration, and favored M2 polarization in an autocrine/paracrine fashion, thus protecting neurons from OGD/R injury [21, 37, 38]. One limitation is that we did not explore the direct effects of Ac2-26 on *in vitro* macrophage polarization. However, this notion may be supported by previously reported studies on ANXA1-mediated inflammatory macrophage polarization in other diseases [39–42]. In addition, Ferraro et al. [43] reported that ANXA1 drives cardiac macrophage polarization toward a pro-angiogenic type and contribute to control myocardial angiogenesis. It should be pointed out that the present study, like the overwhelming majority of other studies, used a binary M1/M2 polarization paradigm [44]. However, it is now increasingly recognized that the concept of M1/M2 polarization is an *in vitro* construction, and only reflects the polarized extremes, which may not be representative of an *in vivo* scenario [45, 46]. Indeed, existing evidence suggests that microglia/macrophages show complex and mixed phenotypes; thus, M1/M2 dichotomy is oversimplified and is inadequate to define the microglial/macrophage polarization states and subsequent inflammatory profiles in cerebral I/R injury. Deng et al. [47] demonstrated that the majority of microglial phenotypes after cerebral I/R injury go beyond the classical M1/M2 categories. Despite these critiques, this distinction may still have important implications for comprehending the role of microglia/macrophages in the process of cerebral I/R injury. At the same time, the heterogeneity of microglial/macrophage and the exact roles of ANXA1 and Ac2-26 in microglial/macrophage polarization warrant further studies. In addition, it is worth noting that Ac2-26 might directly target brain microvascular endothelial cells to exert its protective roles in BBB disruption, because it has been shown that ANXA1 maintains the BBB integrity by stabilizing cytoskeleton and tight and adherens junctions in many neurological disorders [48, 49].

FPR2/ALX receptor, mainly expressed on phagocytes including microglia, is involved in host defense and inflammation. When binding to endogenous anti-inflammatory/pro-resolving ligands (e.g., specialized pro-resolving lipid mediators lipoxin A4 and resolvin D1, and ANXA1) or pro-inflammatory ligands (e.g., formylated bacterial peptides and amyloid beta), FPR2/ALX receptor is activated and triggers downstream signaling cascades and subsequent distinct anti- or pro-inflammatory responses [50, 51]. The immune regulation roles of ANXA1 and Ac2-26 through functional interaction with FPR2/ALX receptor have been reported using pharmacological and genetic approaches [36, 52–54]. In the current study, we utilized the specific FPR2/ALX antagonist WRW4 and confirmed that Ac2-26 could bias the surface FPR2/ALX receptor of microglia/macrophages to mediate phenotype polarization and inflammatory responses both *in vivo* and *in vitro*. Moreover, the downstream mechanisms of ANXA1-FPR2/ALX system that mediate neuroinflammation inhibition are not fully elucidated, although p38 mitogen-activated protein kinase [52] and extracellular signal-related kinase [53] signaling cascades have been implicated. It has been gradually realized that immunometabolic changes are responsible for functional phenotypes of immune cells [55]. AMPK is an energy-sensing serine/threonine protein kinase that maintains cellular metabolic homeostasis [8]. Recent reports suggest that AMPK activation (phosphorylation of α subunit at thr172) and/or downstream mTOR inhibition are involved in inflammation alleviation and macrophage and/or microglial polarization to the M2-phenotype [56–58]. The present study showed, for the first time, that Ac2-26 increased AMPKα phosphorylation and decreased mTOR phosphorylation in the setting of cerebral I/R, these effects were again abrogated by WRW4. Our results were supported by a previous finding in a skeletal muscle injury model by McArthur et al. [42]. They showed that recombinant human ANXA1 mediated macrophages skewing to M2-phenotype, thus accelerating muscle regeneration through activation of FPR2/ALX receptors and the downstream AMPK signaling cascade. In addition, Li et al. [37] demonstrated that SUMOylated ANXA1 regulated microglial activation and induced phenotype shifts by upregulating the levels of autophagy. Considering the role of AMPK/mTOR pathway in autophagy regulation has been well documented [8], ANXA1-FPR2/ALX system might exert its functions by activating AMPK/mTOR/autophagy signaling. However, to confirm this speculation, autophagy-related molecules should be determined, and selective inhibitors for AMPK and mTOR should also be used in future studies.

## Conclusions

In conclusion, we report for the first time that the decreased ANXA1 in patients with AIS were recovered 2nd–3rd after successful recanalization by EVT, which were positively correlated with clinical outcomes, indicating circulating ANXA1 might be a potential prognostic biomarker. Furthermore, through combined *in vivo* and *in vitro* approaches, we demonstrate that ANXA1 mimetic peptide Ac2-26 protects against cerebral I/R injury by modulating microglial/macrophage switch from the pro-inflammatory M1-phenotype to the anti-inflammatory M2-phenotype, which might be obtained through the functional interaction with FPR2/ALX receptor to activate the downstream AMPK-mTOR pathway. These findings indicate that ANXA1 might be a novel adjunct therapeutic strategy for the management of cerebral I/R injury after successful recanalization. Considering nanoparticle-based delivery of Ac2-26 to the lesion site has been developed [39, 59, 60], the translation of ANXA1 to clinical practice is expected to be possible in the future.

## Supplementary Information

**Additional file 1: Supplemental Table 1.** Baseline characteristics of the participants.

**Additional file 2: Fig. S1.** Ac2-26 ameliorated neurological deficit and BBB disruption, and mediated microglial/macrophage polarization via interaction with FPR2/ALX at 1 d post-tMCAO/R. A Schematic diagram of the experimental design. B Neurological function was evaluated by mNSS test. C Quantitative analyses of EB dye extravasation. D Representative western blotting bands and densitometric quantifications of activated microglial/macrophage marker Iba-1, M1-phenotype markers CD16, and M2-phenotype markers CD206. E qRT-PCR analyses of mRNA expressions of M1-phenotype markers (CD16 and iNOS) and M2-phenotype markers (CD206 and Arg-1). F ELISA analyses of the expressions of a pro-inflammatory cytokine IL-1β (M1-phenotype) and an anti-inflammatory cytokine IL-10 (M2-phenotype). Data were presented as the mean ± SD (B *n* = 10/group; C-F *n* = 6/group), and were analyzed by one-way ANOVA followed by Bonferroni's multiple comparison test. *p < 0.05, **p < 0.01, and ***p < 0.001. ^#^*p* < 0.05, ^##^*p* < 0.01, and ^###^*p* < 0.001.

**Additional file 3: Fig. S2.** Ac2-26 did not affect post-OGD/R BV2 cell viability. BV2 cell viability was measured using a 3-(4,5-Dimethylthiazol-2-yl)-2,5-diphenyltetrazolium bromide (MTT) assay kit (Cat. M1020, Solarbio) according to the manufacturer’s instructions. The absorbance at OD 490 nm was measured using a SpectraMax M5 plate-reader (Molecular Devices). Data were presented as the mean ± SD (*n* = 6/group).

## Data Availability

The datasets used and/or analyzed during the current study are available from the corresponding author on reasonable request.

## Abbreviations

AIS: Acute ischemic stroke
Arg-1: Arginase-1
ANXA1: Annexin A1
AMPKα: Adenosine 5’-monophosphate-activated protein kinase α subunit
ANOVA: One-way analysis of variance
BBB: Blood–brain barrier
CD: Cluster of differentiation
CCA: Common carotid artery
CBF: Cerebral blood flow
CCD: Charge-coupled device
CM: Conditioned medium
DMEM: Dulbecco’s modified Eagle’s medium
DAPI: 4’,6-Diamidino-2-phenyl-indole
EVT: Endovascular thrombectomy
ELISA: Enzyme-linked immunosorbent assay
ECA: External carotid artery
EB: Evans blue
FPR2/ALX: Formyl peptide receptor type 2/lipoxin A4 receptor
FPA: Paraformaldehyde
I/R: Ischemia–reperfusion
iNOS: Inducible nitric oxide synthase
IL: Interleukin
ICA: Internal carotid artery
Iba-1: Ionized calcium-binding adapter molecule-1
IQR: Interquartile range
LDH: Lactate dehydrogenase
LVO: Large vessel occlusion
mTICI: Modified treatment in cerebral infarction
mRS: Modified Rankin Scale
mNSS: Modified neurological severity score
mTOR: Mammalian target of rapamycin
NeuN: Neuronal nuclei
NIH: National Institute of Health
OGD/R: Oxygen–glucose deprivation and reoxygenation
OD: Optical density
PBS: Phosphate-buffered saline
qRT-PCR: Quantitative real-time polymerase chain reaction
RT: Room temperature
SD: Standard deviation
tMCAO/R: Transient middle cerebral artery occlusion/reperfusion
TUNEL: Terminal deoxynucleotidyl transferase–mediated dUTP nick end labelling
TBST: Tris-buffered saline containing Tween-20
WRW4: Trp-Arg-Trp-Trp-Trp-Trp-NH2
