# Supplementary material for: Annexin A1 protects against cerebral ischemia–reperfusion injury by modulating microglia/macrophage polarization via FPR2/ALX-dependent AMPK-mTOR pathway
Source: J Neuroinflammation. 2021 May 22;18:119. doi: 10.1186/s12974-021-02174-3 (PMC8140477; doi:10.1186/s12974-021-02174-3)
Supplement: Supplementary file 1 — Additional file 1: Supplemental Table 1. Baseline characteristics of the participants. [file 12974_2021_2174_MOESM1_ESM.docx]

Supplemental Table 1. Baseline characteristics of the participants

| Characteristic | Healthy Controls (n=12) | Total AIS Patients (n=23) | *p* Value | Total AIS Patients (n=23) | | *p* Value |
| --- | --- | --- | --- | --- | --- | --- |
|  |  |  |  | Favorable outcome (n=11) | Unfavorable outcome (n=12) |  |
| Age, year, mean ± SD | 63.83 ± 13.13 | 68.96 ± 9.76 | 0.200^a^ | 65.00 ± 8.22 | 72.58 ± 9.96 | 0.061^a^ |
| Gender, male/female, n | 6/6 | 13/10 | 0.713^b^ | 8/3 | 5/7 | 0.214^c^ |
| Vascular Risk factors, n (%) |  |  |  |  |  |  |
| Hypertension | 4 (33.33%) | 15 (65.22%) | 0.072^b^ | 6 (54.55%) | 9 (75%) | 0.400^c^ |
| Diabetes | 3 (25%) | 5 (21.74%) | 1.000^c^ | 1 (9.09%) | 4 (33.33%) | 0.317^c^ |
| Dyslipidemia | 1 (8.33%) | 2 (8.7%) | 1.000^c^ | 1 (9.09%) | 1 (8.33%) | 1.000^c^ |
| Coronary artery disease | 4 (33.33%) | 8 (34.78%) | 1.000^c^ | 3 (27.27%) | 5 (41.67%) | 0.667^c^ |
| Atrial fibrillation | 0 (0%) | 9 (39.13%) | 0.015^c^ | 4 (36.36%) | 5 (41.67%) | 1.000^c^ |
| Smoking | 2 (16.67%) | 4 (17.39%) | 1.000^c^ | 2 (18.18%) | 2 (16.67%) | 1.000^c^ |
| Admission NIHSS score, mean ± SD | NA | 17.48 ± 8.78 | - | 15.18 ± 10.22 | 19.58 ± 7.01 | 0.238^a^ |
| Stroke location, n (%) |  |  |  |  |  |  |
| Internal carotid artery | NA | 12 (52.17%) | - | 6 (54.55%) | 6 (50%) | 0.827^b^ |
| Middle cerebral artery | NA | 11 (48.83%) | - | 5 (45.45%) | 6 (50%) |  |
| Stroke cause (TOAST), n (%) |  |  |  |  |  |  |
| Cardioembolic | NA | 4 (17.39%) | - | 1 (9.09%) | 3 (25%) | 0.784^c^ |
| Large artery atherosclerosis | NA | 17 (73.91%) | - | 9 (81.82%) | 8 (66.67%) |  |
| Unknown | NA | 2 (8.7%) | - | 1 (9.09%) | 1 (8.33%) |  |
| Medications, n (%) |  |  |  |  |  |  |
| Antiplatelet | 2 (16.67%) | 1 (4.35%) | 0.266^c^ | 1 (9.09%) | 0 (0%) | 0.478^c^ |
| Anticoagulation | 0 (0%) | 2 (8.7%) | 0.537^c^ | 1 (9.09%) | 1 (8.33%) | 1.000^c^ |
| IV rt-PA | NA | 17 (73.91%) | - | 9 (81.82%) | 8 (66.67%) | 0.640^c^ |
| Puncture to reperfusion, min, mean ± SD | NA | 89.05 ± 27.24 | - | 96.89 ± 29.13 | 82.00 ± 24.75 | 0.245^a^ |
| mTICI 2b-3, n (%) | NA | 23 (100%) | - | 11 (100%) | 12 (100%) | - |
| sICH, n (%) | NA | 7 (30.43%) | - | 3 (27.27%) | 4 (33.33%) | 1.000^c^ |

Note: ^a^Analysed by Independent sample t-test; ^b^Analysed by Chi-square test; ^c^Analysed by Fisher’s exact test.

Abbreviations: AIS, acute ischemic stroke; SD, standard deviation; NIHSS, National Institutes of Health Stroke Scale; TOAST, Trial of Org 10172 in Acute Stroke Treatment; IV, intravenous; rt-PA, recombinant tissue plasminogen activator; mTICI, modified Treatment in Cerebral Infarction; sICH, symptomatic intracerebral hemorrhage.
