# Supplementary material for: Annexin A1 protects against cerebral ischemia–reperfusion injury by modulating microglia/macrophage polarization via FPR2/ALX-dependent AMPK-mTOR pathway
Source: J Neuroinflammation. 2021 May 22;18:119. doi: 10.1186/s12974-021-02174-3 (PMC8140477; doi:10.1186/s12974-021-02174-3)
Supplement: Supplementary file 2 — Additional file 2: Fig. S1. Ac2-26 ameliorated neurological deficit and BBB disruption, and mediated microglial/macrophage polarization via interaction with FPR2/ALX at 1 d post-tMCAO/R. A Schematic diagram of the experimental design. B Neurological function was evaluated by mNSS test. C Quantitative analyses of EB dye extravasation. D Representative western blotting bands and densitometric quantifications of activated microglial/macrophage marker Iba-1, M1-phenotype markers CD16, and M2-phenotype markers CD206. E qRT-PCR analyses of mRNA expressions of M1-phenotype markers (CD16 and iNOS) and M2-phenotype markers (CD206 and Arg-1). F ELISA analyses of the expressions of a pro-inflammatory cytokine IL-1β (M1-phenotype) and an anti-inflammatory cytokine IL-10 (M2-phenotype). Data were presented as the mean ± SD (B n = 10/group; C-F n = 6/group), and were analyzed by one-way ANOVA followed by Bonferroni's multiple comparison test. *p < 0.05, **p < 0.01, and ***p < 0.001. #p < 0.05, ##p < 0.01, and ###p < 0.001. [file 12974_2021_2174_MOESM2_ESM.docx]

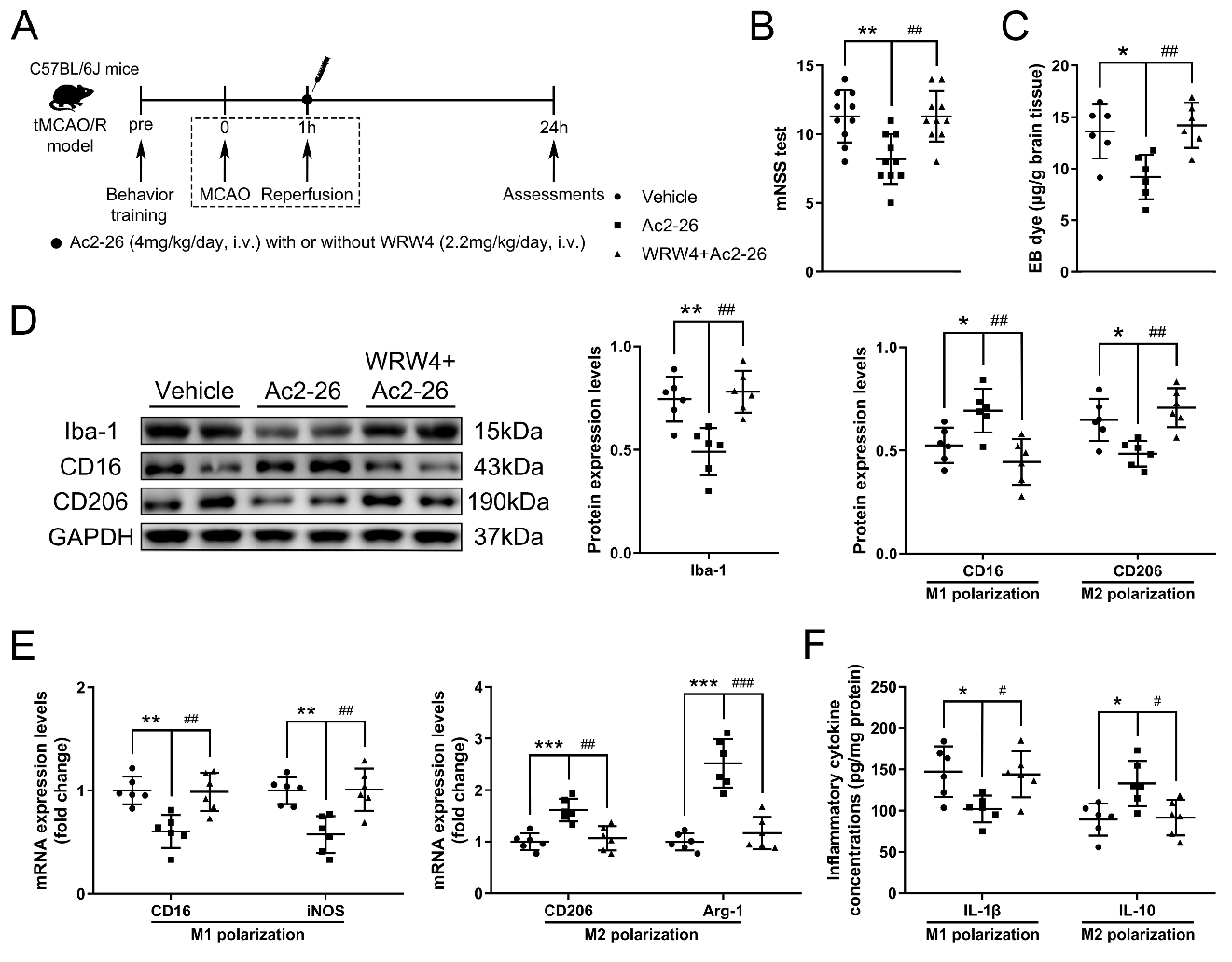


**Fig. S1 Ac2-26 ameliorated neurological deficit and BBB disruption, and mediated microglial/macrophage polarization via interaction with FPR2/ALX at 1 d post-tMCAO/R. A** Schematic diagram of the experimental design. **B** Neurological function was evaluated by mNSS test. **C** Quantitative analyses of EB dye extravasation. **D** Representative western blotting bands and densitometric quantifications of activated microglial/macrophage marker Iba-1, M1-phenotype markers CD16, and M2-phenotype markers CD206. **E** qRT-PCR analyses of mRNA expressions of M1-phenotype markers (CD16 and iNOS) and M2-phenotype markers (CD206 and Arg-1). **F** ELISA analyses of the expressions of a pro-inflammatory cytokine IL-1β (M1-phenotype) and an anti-inflammatory cytokine IL-10 (M2-phenotype). Data were presented as the mean ± SD (B n = 10/group; C-F n = 6/group), and were analyzed by one-way ANOVA followed by Bonferroni's multiple comparison test. *p < 0.05, **p < 0.01, and ***p < 0.001. ^#^p < 0.05, ^##^p < 0.01, and ^###^p < 0.001.
