# Supplementary material for: Annexin A1 protects against cerebral ischemia–reperfusion injury by modulating microglia/macrophage polarization via FPR2/ALX-dependent AMPK-mTOR pathway
Source: J Neuroinflammation. 2021 May 22;18:119. doi: 10.1186/s12974-021-02174-3 (PMC8140477; doi:10.1186/s12974-021-02174-3)
Supplement: Supplementary file 3 — Additional file 3: Fig. S2. Ac2-26 did not affect post-OGD/R BV2 cell viability. BV2 cell viability was measured using a 3-(4,5-Dimethylthiazol-2-yl)-2,5-diphenyltetrazolium bromide (MTT) assay kit (Cat. M1020, Solarbio) according to the manufacturer’s instructions. The absorbance at OD 490 nm was measured using a SpectraMax M5 plate-reader (Molecular Devices). Data were presented as the mean ± SD (n = 6/group). [file 12974_2021_2174_MOESM3_ESM.docx]

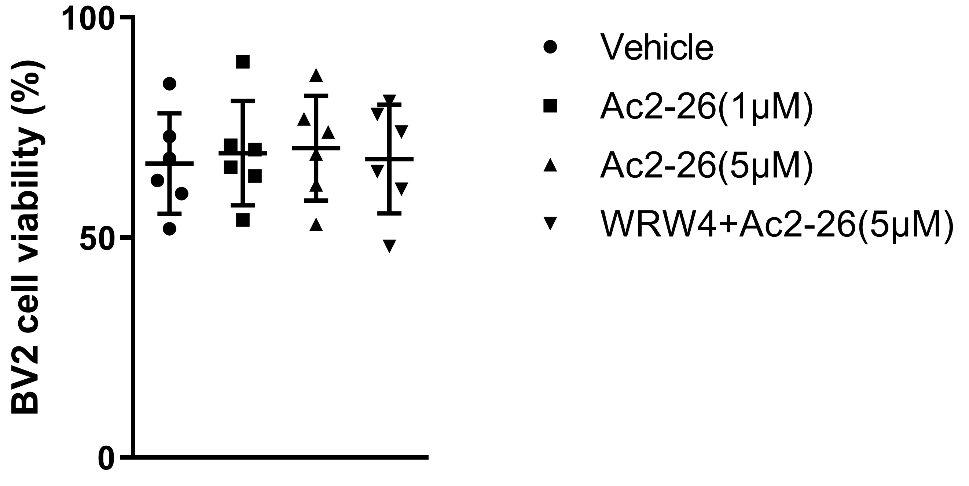


**Fig. S2 Ac2-26 did not affect post-OGD/R BV2 cell viability.** BV2 cell viability was measured using a 3-(4,5-Dimethylthiazol-2-yl)-2,5-diphenyltetrazolium bromide (MTT) assay kit (Cat. M1020, Solarbio) according to the manufacturer’s instructions. The absorbance at OD 490 nm was measured using a SpectraMax M5 plate-reader (Molecular Devices). Data were presented as the mean ± SD (n = 6/group).
